# New technique for the assessment of the growth capacity and development of fetal lungs under compressive circumstances using MRI and 3D models

**DOI:** 10.1590/0100-3984.2022.0025

**Published:** 2022

**Authors:** Pedro Teixeira Castro, Edward Araujo Júnior, Jorge Lopes, Gerson Ribeiro, Heron Werner

**Affiliations:** 1 Biodesign Laboratory, Dasa/Pontifícia Universidade Católica do Rio de Janeiro (PUC-Rio), Rio de Janeiro, RJ, Brazil; 2 Department of Obstetrics, Escola Paulista de Medicine da Universidade Federal de São Paulo (EPM-Unifesp), São Paulo, SP, Brazil; 3 School of Medicine, Universidade Municipal de São Caetano do Sul (USCS), Campus Bela Vista, São Paulo, SP, Brazil

## INTRODUCTION

Bronchial atresia is a rare condition characterized by the absence of communication between the distal bronchi and proximal airway and can affect any segment of the bronchial tract. The severity of malformation is related to the site of obstruction of the proximal airways. Segmental obstruction affects a small volume of the lungs and is usually related to late incidental diagnosis in children and adults^(1)^. Obstruction of the mainstem or proximal lobar bronchus is a rare condition in intrauterine life and in neonates, in whom it causes respiratory difficulties. Such obstruction usually results in severe, lethal conditions^(2)^. The live birth of a neonate with bronchial atresia who thrives with the anatomical repercussions of the malformation and survives the surgical treatment after birth is even more uncommon, only one case having been described.

Ultrasound is the gold-standard method for the diagnosis of bronchial atresia. However, in late pregnancy, polyhydramnios is common due to bronchial atresia-induced mediastinal deviation, which limits the accuracy of ultrasound. Magnetic resonance imaging (MRI) can be a helpful method, adding information regarding the spatial relationship between the enlarged affected lung and the adjacent organs and overcoming the limitations imposed by the increased quantity of amniotic fluid.

## TECHNIQUE

**Figure 1:**
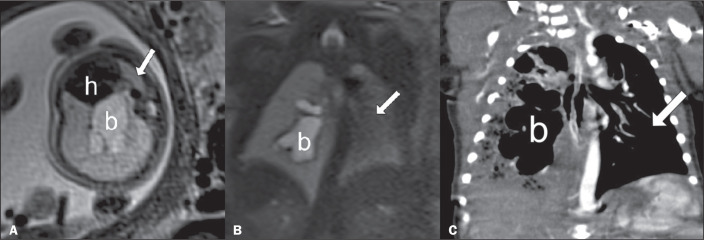
T2-weighted MRI sequences of a fetus with bronchial atresia and a CT scan of the resulting neonate. A: At 25 weeks of gestation, the fetus presented an inflated right lung deviating the mediastinum; the bronchocele (b) is clearly visible at the center of the right lung, and the left lung is compressed and shows low signal intensity (arrow). B: A sagittal image acquired at 35 weeks of gestation, showing less pronounced mediastinal deviation and persistence of the bronchocele (b); the left lung is also visible (arrow). C: A postnatal CT image of the neonate obtained in the second week of life, showing the bronchocele (b) and that the right lung was still causing mediastinal deviation; the left lung (arrow) presents good development. (h, heart).

Fetal MRI can improve the diagnostic accuracy of prenatal imaging, offering high spatial and contrast resolution^(3)^. Images can also be acquired in three-dimensional (3D) sequences, which makes it possible to segment regions of interest for the planning of surgical interventions and for the counseling of parents. The 3D images acquired can be exported to external software, which can make them more comprehensible by better demonstrating the spatial relationships between malformations and the surrounding organs and tissues^(4)^. Here, we present a case of right-sided mainstem bronchial atresia in which the images from prenatal MRI and postnatal computed tomography (CT) were used in order to demonstrate the capacity of a compressed lung to grow over the course of a pregnancy. The images presented (Figure 1) were acquired at 25 and 35 weeks of gestation by fetal MRI, performed in a 1.5-T scanner (Magnetom Aera; Siemens Healthcare, Erlangen, Germany) and in the second week of life by CT (Brilliance; Philips, Solingen, Germany) at a tube current and voltage of 30 mAs and 80 kVp, respectively. They show that the affected lung continued to grow, despite the malformation, and that the contralateral lung grew at an impressive rate, despite the compressive conditions.

**Figure 2:**
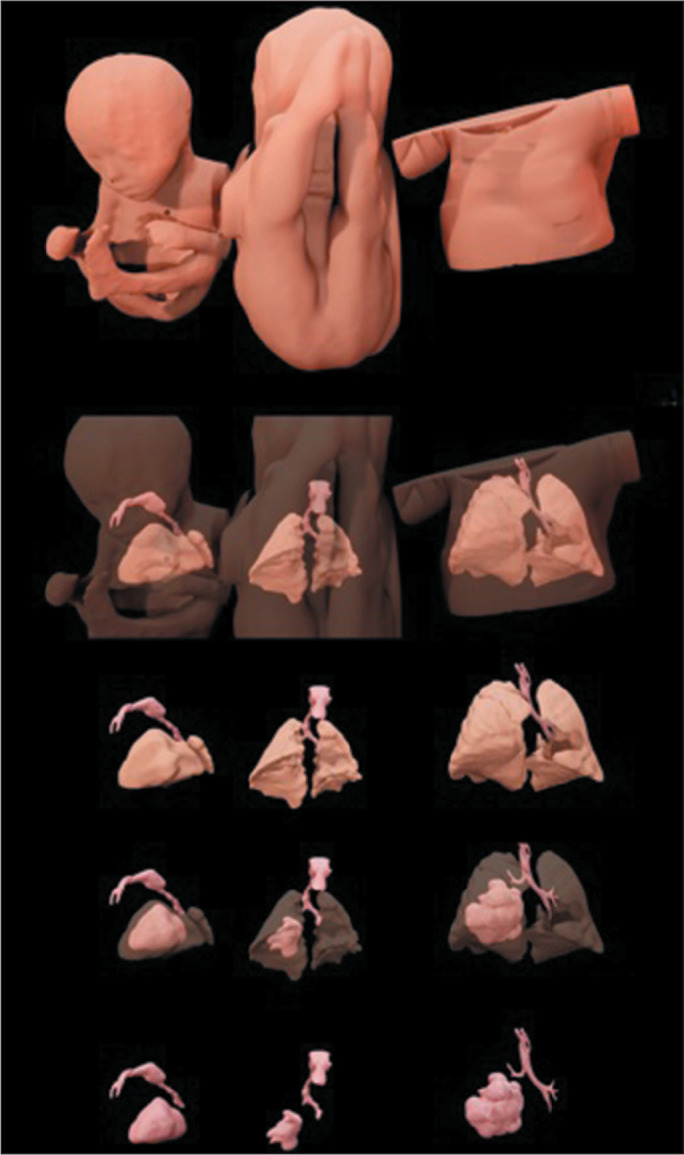
3D reconstructions from fetal MRI scans acquired at 25 weeks of gestation (left column), fetal MRI scans acquired at 35 weeks of gestation (middle column), and CT scans obtained in the second week of life (right column). At 25 weeks of gestation, the bronchocele resulted in enlargement of the right lung, which therefore compressed the left lung. At 35 weeks of gestation, the left lung showed impressive growth in relation to its size at 25 weeks. In the second week of life, the right and left lungs showed good proportionality, a slight mediastinal deviation still was present, and it was possible to identify the right upper lobe.

The images from T2-weighted true fast imaging sequences and CT scans were exported into Digital Imaging and Communications in Medicine files. The fetus, lungs, and bronchocele were manually segmented with the 3ds Max 2019 software package (Autodesk, Mill Valley, CA, USA). The files were then transferred to the MeshLab program, version 2021.07 (Visual Computing Lab, Pisa, Italy), which was used for surface reconstruction and texture mapping, as previously described^(4)^. The final images are shown in Figure 2.

## CONCLUSION

The growth of congenital pulmonary malformations normally peaks around 25 weeks of gestation^(5)^. Using various techniques, we have demonstrated impressive development of the normal lung in a case of mainstem bronchial atresia. The software employed here allows clinical MRI and CT images to be reconstructed in 3D, and the results graphically demonstrate the capacity of the fetal lungs to develop during the second half of pregnancy even under pronounced compression and in severe conditions such as bronchial atresia.
